# Mechanisms of Particles in Sensitization, Effector Function and Therapy of Allergic Disease

**DOI:** 10.3389/fimmu.2020.01334

**Published:** 2020-06-30

**Authors:** Isabella Anna Joubert, Mark Geppert, Litty Johnson, Robert Mills-Goodlet, Sara Michelini, Evgeniia Korotchenko, Albert Duschl, Richard Weiss, Jutta Horejs-Höck, Martin Himly

**Affiliations:** Division of Allergy and Immunology, Department of Biosciences, University of Salzburg, Salzburg, Austria

**Keywords:** adjuvants, alum, animal dander, house dust mite feces, immunomodulation, mold spores, nanomedicine, pollen

## Abstract

Humans have always been in contact with natural airborne particles from many sources including biologic particulate matter (PM) which can exhibit allergenic properties. With industrialization, anthropogenic and combustion-derived particles have become a major fraction. Currently, an ever-growing number of diverse and innovative materials containing engineered nanoparticles (NPs) are being developed with great expectations in technology and medicine. Nanomaterials have entered everyday products including cosmetics, textiles, electronics, sports equipment, as well as food, and food packaging. As part of natural evolution humans have adapted to the exposure to particulate matter, aiming to protect the individual's integrity and health. At the respiratory barrier, complications can arise, when allergic sensitization and pulmonary diseases occur in response to particle exposure. Particulate matter in the form of plant pollen, dust mites feces, animal dander, but also aerosols arising from industrial processes in occupational settings including diverse mixtures thereof can exert such effects. This review article gives an overview of the allergic immune response and addresses specifically the mechanisms of particulates in the context of allergic sensitization, effector function and therapy. In regard of the first theme (i), an overview on exposure to particulates and the functionalities of the relevant immune cells involved in allergic sensitization as well as their interactions in innate and adaptive responses are described. As relevant for human disease, we aim to outline (ii) the potential effector mechanisms that lead to the aggravation of an ongoing immune deviation (such as asthma, chronic obstructive pulmonary disease, etc.) by inhaled particulates, including NPs. Even though adverse effects can be exerted by (nano)particles, leading to allergic sensitization, and the exacerbation of allergic symptoms, promising potential has been shown for their use in (iii) therapeutic approaches of allergic disease, for example as adjuvants. Hence, allergen-specific immunotherapy (AIT) is introduced and the role of adjuvants such as alum as well as the current understanding of their mechanisms of action is reviewed. Finally, future prospects of nanomedicines in allergy treatment are described, which involve modern platform technologies combining immunomodulatory effects at several (immuno-)functional levels.

## The Allergic Immune Response—Basics of the Disease

Worldwide more than a billion people are suffering from allergic disease (1). As one of the most prevalent chronic respiratory illnesses, allergic rhinitis/conjunctivitis and allergic asthma are estimated to affect up to 30% of the population in Western countries (2). Not only the quality of life, but also school and work performance are significantly impacted for those afflicted, further making allergy an economic burden. (3–5). Allergic diseases have seen a dramatic increase; while they have been described as rare in the beginning of the 20th century, it is expected that by 2025, half of the European population will be affected (6, 7). The observed boost in prevalence of respiratory allergy is associated with several factors associated with the “Western lifestyle,” including urbanization, industrialization, agriculture, air pollution, climate change, alterations in biodiversity, increase in personal cleanliness and reduced contact with infectious pathogens (1, 8–10). In this context, this review will on the one hand focus on air pollution, and specifically on particulate matter (PM), which is believed to be among the major factors for the increase in allergic disease prevalences. On the other hand, this review will detail how the resulting disease burden manifests upon exposure to common aeroallergens of grass/weed/tree pollen, house dust mite, pet dander, and mold spores.

According to common text book knowledge (11, 12) the allergic response is divided into two stages: (i) the sensitization phase, which is accompanied by an immune deviation toward a T helper (Th)2-type response and is facilitated by allergen-specific Th2 cells secreting the cytokines interleukin (IL)-4, IL-5, and IL-13, ultimately leading to the generation of allergen-specific immunoglobulin E (IgE) antibodies; in the second stage, (ii) the effector phase, IgE-loaded mast cells (MCs) and basophils degranulate upon exposure to the allergen source, resulting in the release of mediators (i.e., histamines, prostaglandins, leukotrienes). Consequently, individuals with respiratory allergies suffer from symptoms typical for rhinoconjunctivitis, such as a runny nose, sneezing, itching, and watery or swollen eyes. More critical symptoms comprise signs of airway hyperresponsiveness (AHR), characterized by shortness of breath, coughing and wheezing. Allergies are commonly diagnosed *via* clinical anamnesis, skin testing and *in vitro* methods for quantification of allergen-specific IgE (13). Allergy treatment mostly consists of pharmacological interventions with antihistamine, corticosteroids, MC stabilizers, anti-cholinergic agents and leukotriene inhibitors (14). The only curative treatment, however, is allergen-specific immunotherapy (AIT), which displays success rates of around 80% for respiratory allergies (15, 16). While allergen avoidance remains the most important type of intervention, there are notable limitations concerning inhalable environmental allergens in respiratory allergies. As climate change has extended pollen season, higher pollen counts have been documented in several European countries (17). A German study has furthermore observed an association between ozone levels and reactivity to allergen extracts in skin tests (bigger wheal and flare sizes) (18). Likewise, combustion-derived PM is believed to increase allergic reactions by interacting with pollen, as shown by increases in hospital visits related to pollinosis on days with high PM levels (19).

## The Allergenic Entities—Discrepancy Between Molecular Understanding and Clinical Reality

Humans are constantly exposed to allergenic substances in the form of particulates releasing biologically active substances, i.e., proteins and other biomolecules which come into contact with the human mucosal tissue. More than 150 pollen allergens originate from environmental sources such as grasses, weeds, and trees and they have been recognized to play a significant role in triggering allergic responses in sensitized individuals (20, 21). In addition to seasonally confined outdoor allergens, indoor allergens lead to perennial exposure, which has further implications for the clinical outcome in the affected patients (11). The major cat allergen Fel d 1 was detected in 99.9 and 99.7% of American homes, respectively, in two large US surveys (22, 23). Similar findings were reported for the major dog allergen Can f 1 and detectable levels of the individual group 1 and 2 house dust mite (HDM) allergens were found in 60-85% of surveyed homes (23, 24). Fungal spores are ever present and constitute the biggest proportion of aerobiological PM (25), even exceeding pollen grains (26). The official allergen database (www.allergen.org) of the WHO/IUIS currently lists 961 distinct sequences of allergenic molecules, and many more isoallergens and variants, classified into 852 taxonomic groups (www.allergenonline.org). Interestingly, among the 17,929 currently listed protein families (http://pfam.xfam.org/), allergens only appear in 216 Pfam domains, thus, constituting a share of just 1.3% (http://www.meduniwien.ac.at/allfam/). A recent report by the European Academy of Allergy and Clinical Immunology (EAACI) has pointed out discrepancies between the molecular definition of allergic sensitization, in the *Molecular Allergology User's Guide* termed bottom-up approach, and observations made in clinical settings considered as top-down approach (27). Since physicians commonly use natural allergen sources for allergy diagnosis, this implies that the degree of allergenicity is determined not only by the mixture of allergenic proteins itself but by a variety of bystander substances and other co-factors contained in the allergen source. Discrepancy also exists when it comes to the clinical efficacy of allergic treatment by immunotherapy using crude natural extracts or chemically modified preparations, so-called allergoids, *vs*. highly purified recombinant molecules, while safety concerns clearly direct the way into a future of using recombinant allergens enabling development of genetically modified products with optimized safety profiles termed hypoallergens (28, 29). In the present review, we will give a broad overview on the particulate aspects in sensitization, effector function and therapeutic treatment of allergic disease.

## The Role of Particulates in Allergic Sensitization—What are we Exposed to?

**Figure 1:**
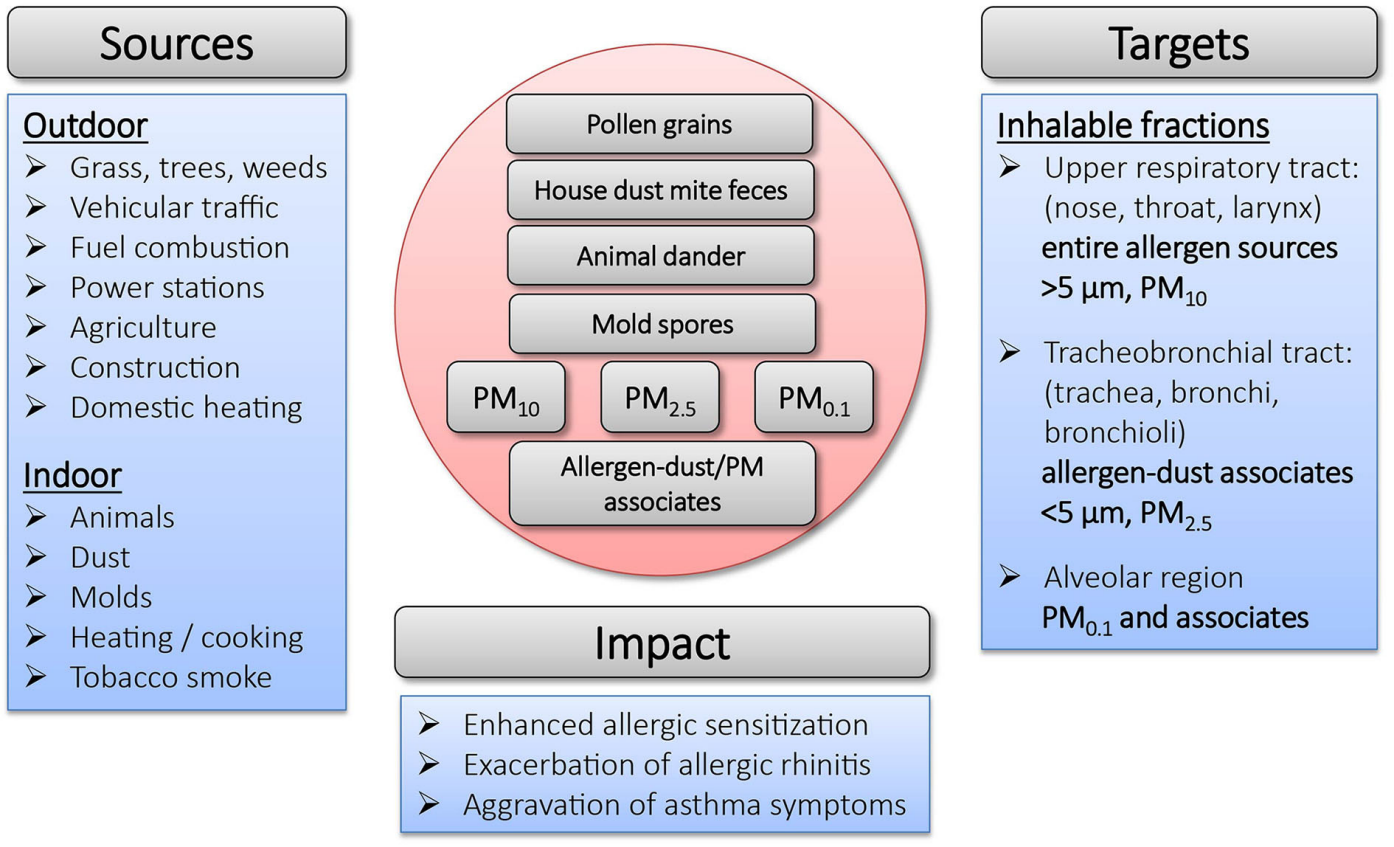
Overview on sources, targets and impact of particulates in allergic sensitization.

Exposure to allergens is not only dependent on their environmental distribution, but also on the form in which they become airborne and their aerodynamic properties. Due to their molecular size and vapor pressure, allergenic proteins and glycoproteins cannot become airborne themselves, but instead are either contained inside particulates (*e.g.*, pollen grains), or attach to airborne particles such as dust (*i.e.*, fragments of human keratin or animal epithelium) (30–32). In general, particle-bound allergens smaller than 5 μm can stay suspended in the air for longer periods of time (33), while larger ones settle quickly (34). An overview on the sources, the targets and the impact of particles on allergic sensitization is depicted in Figure 1.

The major cat allergen Fel d 1 is produced in sebaceous and salivary glands of cats and is found on its fur, skin and in saliva (35). While the primary source of cat allergens is assumed to come from dander (36, 37), Fel d 1 can also be found associated to a range of differently sized dust particles from <1 to 20 μm (38), some of which can remain airborne for 15–35 min after disturbance (33). The highest concentrations of the major HDM allergen Der p 1 is found in mite feces, whose physical properties are similar to those of pollen grains. De Lucca et al. (39) investigated particles containing HDM allergens and reported size ranges of 15–40 μm (feces), 10–150 μm (fibers) and 5–50 μm (flakes). Particles of that size can only be deposited on the nasal mucosa and do not enter the lungs. The prevalence of mold is highly dependent on season and climatic factors (humidity, temperature and wind) and their spores have a wide spectrum of different shapes and sizes in the range of 2–250 μm. A substantial proportion of fungal spores is small enough to enter the lower airways and common allergens are found in the respirable fine particle fraction (<3 μm) (40). While pollen grains themselves are large (10–200 μm) (41), and only few can reach the lower airways, grass, weed and birch pollen allergens associated with particles under 5 μm (starch grains, subpollen particles) have been shown to be released from the pollen grain after light rainfall (42–45). They can furthermore occur in association with smaller airborne fractions such as suspended particulate material deriving from industrial combustion and vehicle exhaust emissions (46, 47). Under occupational settings, mixed aerosols containing food-borne allergens have been identified as sources for a higher prevalence of fish allergy in fish-processing workers (48).

Respirable diesel exhaust, indoor soot and dust particulates especially are known for their capability to bind various classes of allergens *in vitro* and might facilitate their transport into peripheral and deep airways (49–51). Hence, the outcome of allergen exposure is lastly also dependent on the nature of the particle carrying it. Diesel exhaust particles (DEPs), for example, have been shown to contribute to asthma and allergic airway disease (52–55). Binding to carrier particles can furthermore enable allergen concentration, increasing their potential for triggering asthma attacks (49). This may explain the importance of combustion-derived PM in the context of allergic disease, as it provides an ideal vehicle for the distribution and uptake of allergens, which would otherwise not reach the lower airways. Furthermore, PM also displays the capacity to modify the immunological reactions against the transported allergen. The latter will be discussed in detail in this review.

For environmental studies of air pollution, PM is commonly divided according to its size (aerodynamic diameter) into different categories: PM_10_, coarse particles with an aerodynamic diameter ≤ 10 μM, PM_2.5_, fine particles with a diameter ≤ 2.5 nm and PM_0.1_, ultrafine particles with a diameter ≤ 0.1 μm (56). The size of PM is a crucial factor concerning its capability to penetrate into the respiratory tract: while PM_10_ is only able to reach the upper respiratory tract, PM_2.5_ reaches the tracheobronchial tract and PM_0.1_ is able to penetrate into the alveolar region (57). The composition of PM varies among geography, season and proximity to roadways (58). Various studies have shown that PM exposure is associated with enhanced allergic sensitization and aggravation of asthmatic symptoms (59, 60). Moreover, the exposure to PM during childhood is believed to contribute to the increase in allergies worldwide (61). In the context of allergic disease, PM has furthermore been shown to exert harmful effects as a chemical toxin. Upon inhalation, PM can induce cell stress and toxicity, dependent on its particle size, chemical composition, and surface-bound molecules (62). For instance, PM containing transition metals such as iron have been shown to exert genotoxic effects and increase the production of reactive oxygen species (ROS) (63, 64). Furthermore, PM-associated endotoxins can contribute to increased airway inflammation and dysfunction (65) In summary, PM has differential implications on allergic disease by either enhancing allergic sensitization or exacerbating pulmonary symptoms, as will be discussed in further detail in subsequent sections of this review.

Outdoor PM can be derived from various sources such as vehicular traffic, fuel combustion, agriculture or industry, but also non-anthropogenic sources like volcanic eruptions, wildfires or ocean-derived salts. A major component of traffic-related outdoor PM are DEPs, which typically consist of a carbon core that has adsorbed different organic compounds, *e.g.*, polycyclic aromatic hydrocarbons (PAHs), transition metals and other compounds (66, 67). The combined effects of air pollution and pollen grains on cells, animal models and allergic patients have been extensively reviewed (68). Different types of particulates were found to be adsorbed to pollen grains (69). DEP-associated PAHs have been shown to exert pro-allergic effects on basophils of birch pollen-allergic patients in an allergen-independent manner (70). Another study has shown that DEPs disrupt the nasal epithelium and thereby lead to the exacerbation of allergic rhinitis symptoms in a mouse model (71). The PIAMA prospective birth cohort study has identified metal constituents of non-tailpipe road traffic emissions such as iron, copper and zinc as risk factors for respiratory disease in school children (72).

Indoor PM is an important factor as most people spend 90% of their time indoors (73). A major source of indoor PM is tobacco smoke. It accounts for around 50–90% of the total indoor PM concentration in areas frequented by smokers (57). The particles sizes present in smoke caused by six different commercial available cigarettes was analyzed by Becquemin and co-workers and determined to be 0.27 ± 0.03 μm (inhaled by the smoker) or 0.09 ± 0.01 μm (inhaled by passive smokers) (74). Besides its well-known risk for causing lung diseases in smokers as well as in non-smokers who are exposed to second-hand smoke (75), there is evidence that environmental tobacco smoke is responsible for an increased sensitivity to allergens in children (76). In a birth cohort study, Thacher et al. (77) found that maternal smoking during pregnancy did not relate to sensitization to food allergens. However, exposure to parental smoking during early infancy was shown to increase the risk of food allergen sensitization during childhood and adolescence. Contrasting results were obtained in studies by Shargorodsky et al. (78) who showed that tobacco smoke exposure was related to increased prevalence of rhinitis symptoms, but independent from allergic sensitization in US adults. In another study, a decreased prevalence of allergic sensitization of children was found in respect to tobacco smoke exposure (79). The connection of tobacco smoke and allergy may thus be complex, but it is clear that smoking interferes with immunity at different levels. A review by Maes and colleagues describes that both, tobacco smoke and DEPs, affect allergic sensitization and the development or exacerbation of asthma by similar mechanisms (80) suggesting that the particular characteristics of combustion-derived PM can play an important role here.

Co-exposure to PM and specific allergic sensitizers such as pollen, HDM feces, mold spores or animal dander is difficult to study in humans under real-life environmental conditions. The main problem is that sensitization is a highly individual response. Its investigation relies on application of *in vitro* methods or use of *in vivo* animal models, which is also how co-exposure has been experimentally addressed (57). A study by Acciani et al. (81) revealed that young BALB/c mice display intensified features of allergic sensitization, including an increase of IgE, inflammatory cells, and Th2/Th17 cytokines after co-exposure to DEPs and HDM, while DEPs alone in the same concentration did not lead to the aforementioned effects. A later study by the same researchers reported that combined exposure to DEPs and HDM leads to a significantly higher number of specific memory T cells in the murine lung promoting secondary responses to the allergen (82). The authors also showed a prolonged presence of DEPs in lung macrophages, but excluded DEPs as the culprits of increased HDM recall responses observed in lymphoid organs. This study illustrates the difficulty in understanding the detailed mechanism and role of particles in the context of allergic disease. Nevertheless, it is clear that DEPs in combination with allergen lead to an exacerbation of the Th2-driven response compared to the effects caused by the allergen alone. The authors, furthermore, proved relevance of their mechanistic investigations in mice for humans by analyzing the Cincinnati Childhood Allergy and Air Pollution Study birth cohort resulting in positive association of increased asthma prevalence in allergic children with early-life exposure to high DEP levels compared to non-allergic children. Castaneda and colleagues strengthened these mechanistic findings by showing that PM enhanced allergic immune responses of Balb/c mice, characterized by increased monocyte and eosinophil migration, Th2 cytokines and IgE expression (83). They, furthermore, suggested that PAH contents are responsible for Th17 immune responses by activation of the aryl hydrocarbon receptor. Taken together, it is likely that both particle-specific effects as well as effects exerted by PM-associated chemicals play a role in the enhanced immune responses observed upon co-exposures.

## Immune Cells Involved in Allergic Sensitization—How do They React to Particles?

The mechanisms underlying allergic sensitization in general, and upon exposure to particulate matter, are still not fully understood. Recently it has been shown that sensitization toward certain allergens may not primarily result from intrinsic properties of the allergen itself, but can also depend on immunomodulatory compounds co-delivered with the allergen (84, 85). To better understand the complex process of allergic sensitization, the following section gives a brief overview on the different types of immune cells, their interactions, and their possible role as triggers of particle-mediated allergic reactions.

**Figure 2:**
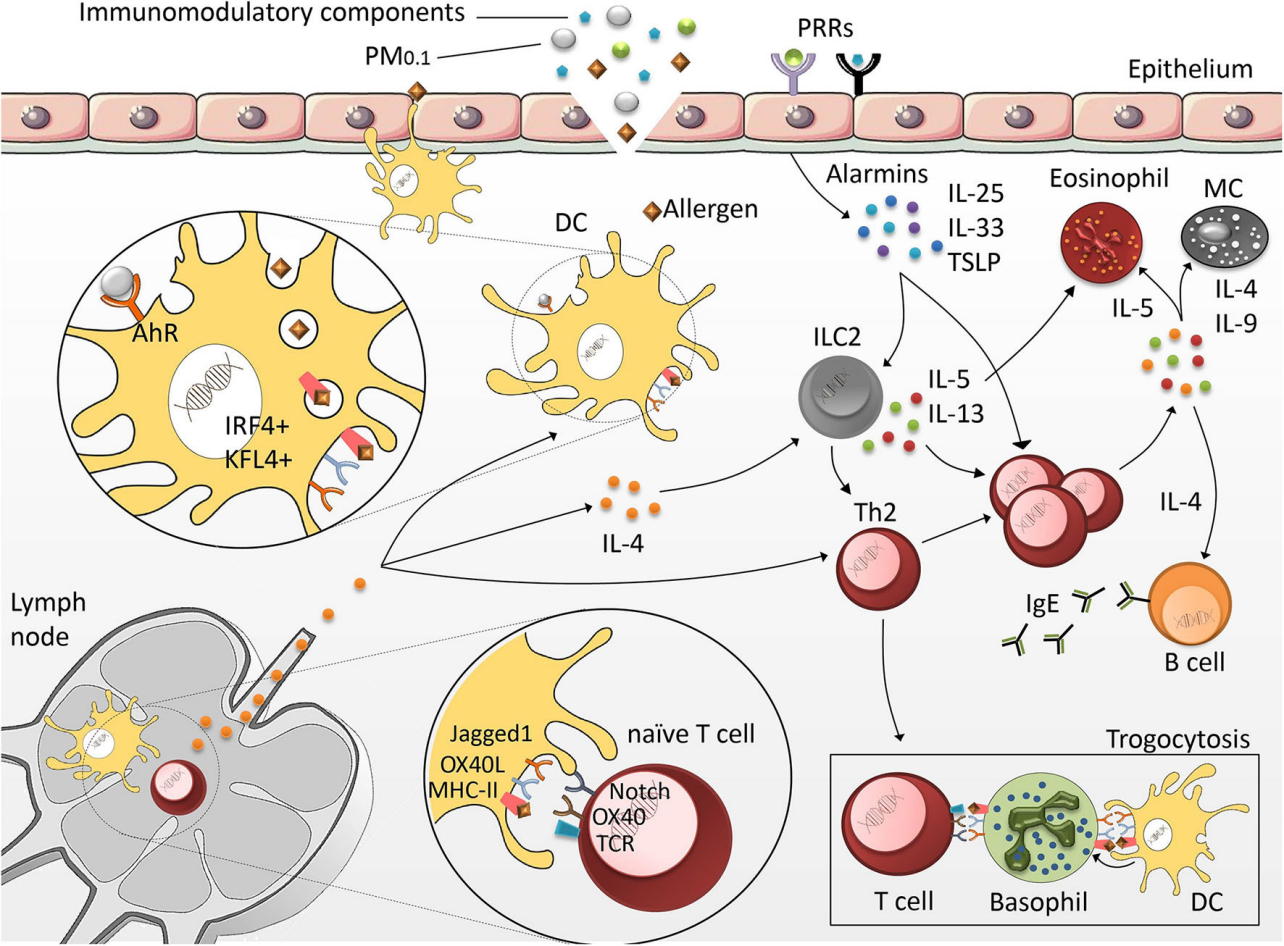
Mechanisms of allergic sensitization. DCs located in the epithelia of peripheral tissues are exposed to allergens, immunomodulatory compounds and particulate matter (PM). Once activated, highly specialized CD11b^+^/CD301^+^/PDL2^+^/KFL4^+^/IRF4^+^ DCs migrate into the lymph nodes to initiate the Th2 differentiation program. Upon exposure to epithelial-derived factors (IL-25, thymic stromal lymphopoietin (TSLP) and IL-33), group 2 innate lymphoid cells (ILC2) produce Th2 cytokines. This further promotes Th2 cell polarization and leads to exacerbation of the allergic response characterized by the secretion of IgE by B cells, and the activation of MCs and eosinophils. Basophils act as antigen-presenting cells (APCs) by presenting DC-derived antigenic peptides to T cells *via* a specific membrane transfer mechanism called trogocytosis.

Dendritic cells (DCs) play a crucial role in priming specific T cell responses (Figure 2). They are located close to the epithelial barriers, where they are exposed to allergens, allergen-associated immunomodulatory components and particulate matter. Once activated by the respective stimulus, DCs undergo a specific maturation process, which primes them to promote the differentiation of naïve CD4^+^ T cells into Th2 cells (86). While DCs can release T cell-priming cytokines that determine the type of immune responses, IL-4, the classical Th2-priming cytokine, is not produced by DCs. This raises the question whether, in the absence of alternative Th cell-priming stimuli, DCs would induce a Th2 phenotype by default. Another hypothesis states that DC-derived stimuli, other than IL-4, can potently induce Th2 differentiation as well. One example are DCs, which are characterized by the expression of CD11b^+^ CD301b^+^ PDL2^+^. These cells are particularly potent activators of Th2 cell differentiation and further enhance their Th2-polarizing capacity by expression of the costimulatory molecules OX40L and Jagged 1 (87–90). Especially upon exposure to fine particles and ultrafine particles, the Jagged 1/Notch signaling axes is essential for allergic inflammation (91). Jagged 1 interacts with Notch receptors on T cells, which can promote Th2 cell polarization *via* induction of GATA-3 (92, 93). This mechanism is particularly important in PM_0.1_-induced allergic inflammation of the airways. PM_0.1_-dependent transcriptional expression of Jagged 1 (91, 94) seems to be regulated *via* the aryl hydrocarbon receptor, which in turn is activated by PAH contained in the particles. Thus, PM_0.1_-induced Jagged 1 transcription depends on aryl hydrocarbon receptor, but is independent of classical pattern recognition *via* Toll-like receptor (TLR)4 and NOD-like receptors (NLRs) (94). Neutralizing Notch-signaling downstream of Jagged 1/Notch interactions further demonstrated that PM_0.1_-induced exacerbation of allergic airway inflammation was abrogated (91). This points toward a crucial involvement of the Jagged/Notch pathway in promoting particle-mediated Th2 immune responses.

In addition to OX40L and Jagged 1, it was shown that DCs capable of inducing Th2 responses also require Interferon regulatory factor 4 (IRF4) and Krüppel-like factor 4 (KLF4). Mice with IRF4 deficiency in the DC lineage show a strongly reduced population of CD11b^+^ CD301b^+^ PDL2^+^ DCs, which correlates with a strong reduction of allergen-induced lung inflammation (95, 96). Conditional KLF4 deletion within conventional CD8α^+^-type DCs provided evidence that KLF4 is required to promote Th2 cell responses induced upon HDM challenge or helminth infection, whereas KLF4 deletion did not affect Th1 or Th17 responses in other infection models (97). These experiments suggest that KLF4 is an important molecule enabling DCs to promote Th2 immunity.

Although DCs are the most important cell type contributing to the development of type 2 responses, naïve Th cells also receive important signals from epithelial barrier organs. Allergen contact, protease activity of certain allergens, and cell damage can result in the release of cytokines and alarmins including IL-25, thymic stromal lymphopoietin (TSLP) and IL-33 from epithelial cells (98). Th2 cells upregulate the receptors for these cytokines, indicating that priming as well as re-activation of Th2 cells is strongly promoted by those factors (99, 100). Especially at sites of inflammation, IL-25, IL-33, and TSLP facilitate terminal differentiation of Th2 cells and foster their effector functions (101).

The inability of DCs to produce IL-4 has drawn the attention to basophils, which produce substantial amounts of IL-4. Murine basophils additionally express MHC class II as well as the co-stimulatory molecules CD80 and CD86, indicating that these cells might be able to initiate the process of Th2 differentiation (102). However, human studies failed to confirm the important role of basophils in priming Th2 differentiation (103, 104) and later studies have shown that inflammatory CD11b^+^ conventional DCs, rather than basophils, are crucial for the initiation of Th2 responses (105). The role of basophils in priming the Th2 response is now explained *via* trogocytosis. This is a process through which cells (in this case basophils) extract membrane fragments from neighboring cells (in this case DCs), thereby passively acquiring peptide-MHC class II molecules from DCs, to control Th2 development as antigen-presenting cells (APCs) (106).

Group 2 innate lymphoid cells (ILC2s) also play an important role in the induction and maintenance of immune responses mediated by Th2 cytokines. Since ILC2s do not express rearranged antigen receptors or pattern recognition receptors, they are mainly activated by signals derived from the epithelial barrier, including IL-25, IL-33, and TSLP (107). Similar to Th2 cells, ILC2s are characterized by high expression of the transcription factor GATA3 (108), which simulates the transcription of IL-5 and IL-13 (109, 110). In animal models of allergic asthma, ILC2s were identified as the major source of IL-5 or IL-13 (111). Upon stimulation with *Heligmosomoides polygyrus*, ILC2s can also secrete IL-4 in a Leukotriene D4-dependent way. Specific deletion of IL-4 from the ILC2 compartment abrogated the *Heligmosomoides polygyrus*-induced Th2 response, indicating that ILC2-derived IL-4 was sufficient to promote a Th2 response in this model (112). Thus, synergistic effects between ILC2s and Th2 cells may be required to achieve maximum release of Th2 cytokines.

## Aggravation of Allergic Asthma by Inhaled Aerosols

Asthma can generally be described as hyperresponsiveness and obstruction of the airways caused by chronic inflammation and an overproduction of mucus (113). Although asthma tends to be a lifelong condition, its severity can vary throughout the patient's life. Chronic inflammation is facilitated by the infiltration of a collection of inflammatory cells including eosinophils, MCs and CD4^+^ T cells (114). While T cells are known to express Th2-type cytokines associated with the aggravation of asthma symptoms (115–117), they can also play a role in regulating the Th2 response and hence, alleviate allergic diseases (118–122). Worldwide, ~300 million individuals are affected by asthma, with its prevalence increasing over the last decades (123, 124). However, some studies have suggested that the prevalence of asthma might have reached a plateau in Western countries (125–128). Among the multiple clinically identified types of asthma, allergic asthma is the most common (129–131).

Many different external factors have been associated with the exacerbation of asthma symptoms, including viral infections and exposure to air pollutants (53, 132, 133). Immunological effects exerted by particulates heavily depend on the tested material as their immunomodulating properties vary with material chemistry as well as size, shape, and surface-properties (134–137). Consequently, it is essential to evaluate individual particulates and avoid generalizations on the basis of single attributes. Silica NPs have been shown to increase the number of eosinophils found in the bronchoalveolar lavage fluid (BALF) in an ovalbumin (OVA)-asthma model (138) and to raise serum IgE titers (139). Furthermore, enhanced AHR and modulation of inflammatory cytokines and chemokines was observed. This modulation mainly consisted of an increase in the asthma-associated cytokines IL-4, IL-5, and IL-13 in response to the application of OVA-silica NP conjugates and was not observed for OVA alone (140, 141). Carbon NPs have also been shown to affect mouse asthma models. Carbon black NPs given in conjunction with OVA increased inflammatory- and antigen-presenting cell counts in the lung in an OVA-mouse model (142, 143).

It is worth mentioning that silver NPs have been shown to mitigate asthma and decrease the levels of IL-4, IL-5, IL-13, and NF-κB in addition to lowering AHR in OVA-induced allergic inflammation mouse models (144, 145). Although various studies demonstrate the suppression of allergic responses by silver NPs, they have also been shown to increase neutrophilia and levels of circulating TNF-α in an allergen-independent context (146, 147). However, it is important to note that effects observed for silver NPs could, at least in part, be attributed to the dissolved fraction of the material, as Ag^+^ ions are known to be biologically active (148, 149). In the case of lung exposure, different results were observed between silver NPs and Ag^+^ ions using instillation experiments in mice (150). These findings might be further substantiated by a study from Seiffert et al. (151) which implied that interactions with lung surfactant provides a stabilizing effect on the NP surface preventing the release of Ag^+^ ions.

Studies on the effects of gold NPs on asthma show contradicting results and are dependent on the asthma model used. Gold NPs were found to increase AHR and the neutrophil/macrophage count in BALF in a toluene diisocyanate-induced asthma model (152). PEGylated and citrated gold NPs, however, decreased the mucus production, cytokine levels and inflammatory cell accumulation in the lung in an OVA-asthma model (153, 154).

Not only single pristine particle sources can have an effect on asthma exacerbation. It has been shown that ambient air-derived PM_2.5_ can aggravate asthmatic symptoms in an OVA-asthma mouse model. Studies have shown that PM_2.5_ in conjunction with OVA increased the levels of Th2 cytokines, AHR, and the number of eosinophils and neutrophils in BALF (155–157). PM_2.5_ had been obtained by filtering ambient air followed by up-concentration, however, the exact chemical composition was not elucidated. One of the studies attributed the elevated AHR to an increase in apoptosis and TIM-1 activation, which was also witnessed in the OVA/PM_2.5_ group (156). Another study has shown that prolonged exposure to high concentrations of PM_2.5_ leads to an increase in AHR, which was linked to necroptosis-induced neutrophils as well as IL-17 production (158). PM_2.5_ are not only linked to the induction and increase of asthmatic symptoms in animal models, but have also shown to be associated with a higher frequency of emergency room visits upon human exposure to wildfire-related particulate matter (159, 160). Moreover, controlled human exposure studies have shown an association between DEP-allergen exposure and an increase in IL-5, eosinophil cationic protein and airway eosinophils (161). Interestingly, females and adults over the age of 65 years are suggested to be more susceptible to smoke-derived PM_2.5_, indicated by a comparably higher number of ER visits in these cohorts (160).

A possible explanation for the health impacts exerted by particulate matter, and carbon black in particular, is its potential to modify methylation patterns. Sofer et al. (162) for instance, have shown a correlation between carbon black and sulfate particle exposure and changes in methylation patterns in the asthma pathway. The identified affected genes were coding for the high-affinity IgE receptor alpha and gamma subunits, the major basic protein of eosinophil granules, and for IL-9 (162). Nadeau et al. (163) also demonstrated a link between exposure to particulate matter and methylation. In their study, exposure to ambient air pollution was associated with hyper-methylation in the Foxp3 locus, which impairs regulatory T cell (Treg) function and in turn increases asthma morbidity (163).

Additionally, indoor particulates in the fine (PM_2.5_) and coarse (PM_10_) range have been shown to directly affect asthmatic symptoms. This was demonstrated by two different studies, which were able to link elevated levels of indoor PM to an increase in asthmatic symptoms and the use of rescue medication in children (164, 165). Long-term as well as short-term exposure to PM has an impact on the aggravation of asthmatic symptoms. This was illustrated by a study showing a decrease in FEV_1_ (Forced Expiratory Volume in 1 second) and an increase of neutrophilic lung inflammation determined in asthmatic patients already after a 2 h walk along a polluted street in London (166). Moreover, indoor dust biological ultrafine particles, which are mainly composed of microbial extracellular vesicles, have been shown to induce neutrophilic inflammation and, thus, contribute to pathogenesis of chronic lung diseases, such as asthma, chronic obstructive pulmonary disease, and lung cancer (132). In this regard, extracellular vesicles in indoor dust may be recognized as important diagnostic and therapeutic targets. The following sections will first address the allergen-specific and, later, the particle-related aspects of immune deviation as well as the potential of nanomaterials as carrier platforms in allergy treatment.

## Allergen-Specific Immunotherapy

Allergen avoidance and pharmacotherapy aim to build a first line of defense and relieve symptoms of allergy (167–169). However, pharmacotherapy does not prevent allergic disease progression and has to be administered as long as symptoms prevail (170), which typically translates into life-long treatment. The efficacy of allergen avoidance is not supported by robust evidence (171) and is furthermore not feasible in every case (172). So far, AIT is the only curative treatment for allergic diseases as it reinstates immune tolerance against allergens (173).

**Figure 3:**
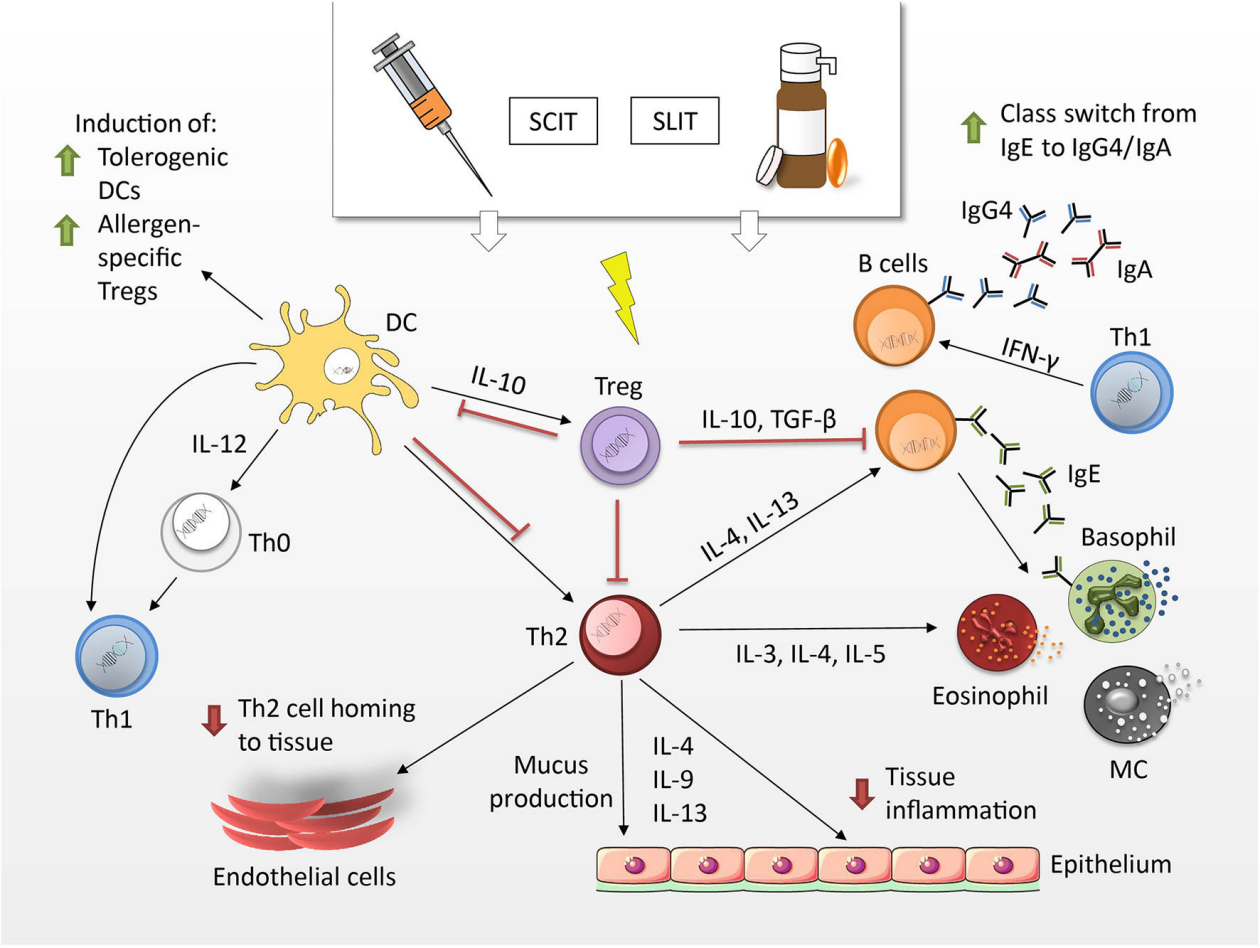
Mechanisms of allergen-specific immunotherapy. Tregs produce cytokines such as IL-10 and TFG-β, which have the potential to suppress Th2 responses. Upon induction of tolerogenic DCs Th1 are mobilized at the expense of Th2 cells. They produce IFN-γ and stimulate the production of IgG4 and IgA antibodies by means of class switching. IgG4 antibodies can block allergen-induced MCs, basophils and eosinophils and hence, limit allergic symptoms by decreasing mediator release (178, 179).

First described by Leonard Noon and John Freeman in the early 20th century (174), AIT is a highly effective treatment for individuals suffering from IgE-mediated diseases (175–177). The primary goal of AIT is the inhibition of both early- and late-phase allergic responses, which are regulated by a plethora of cellular and molecular events (Figure 3). Three main mechanisms are suggested to lead to the induction of tolerance after successful treatment, differentiating AIT from other vaccines: (i) immune deviation toward a Th1-oriented response (and reduction of atopy-associated Th2 responses); (ii) production of allergen-specific IgG4 antibodies and (iii) induction of Tregs and regulatory B cells, (173, 180, 181).

AIT can be administered to adults and children *via* the subcutaneous (SCIT)- or sublingual (SLIT) route. SCIT treatments consist of an initial up-dosing phase, in which increasing doses of allergen are administered to carefully assess the patient's individual sensitivity and the maximum-tolerated dose. This allergen dose is then continued throughout the maintenance phase. Individual shots have to be given with a physician present, due to the risk of adverse effects, which can range from local site reactions to systemic reactions, such as anaphylaxis (182). Sublingual routes for IT have been first proposed in 1986 (183) and facilitate the mucosal deposit of the allergen in the form of drops or dissolving tablets/capsules under the tongue. While this route has been tested in clinical trials in the US, only a few SLIT products have yet been approved by the U.S. Food and Drug Administration (FDA) (184). In general, SLIT is associated with a favorable safety profile, lower risks and offers patients the convenience of an at-home and injection-free administration. It has been suggested that it requires the continuous administration of SCIT/SLIT for 3 years to achieve immunological changes consistent with allergen-specific tolerance in allergic rhinitis, which can be sustained for at least 2 to 3 years after treatment cessation (185).

Although AIT has been well-established and shows successful curation of allergic rhinitis and asthma in many cases, treatments are generally costly, time-consuming and only a few allergens have been standardized for SCIT and SLIT. In recent years, several novel administration routes have been under investigation, including intralymphatic (ILIT), epicutaneous (EPIT), and intradermal (IDIT) routes (186–189). Furthermore, intensive research is focusing on allergoids (*i.e.*, chemically modified/crosslinked allergen particles), allergen-fragments, fusions, hybrids and biological immune response-modifiers for new vaccines to provide a safe, persistent and life-long cure of allergic disease (188, 190–197). In several of the before-mentioned approaches, an interrelation between allergen-specific and particle-related aspects can be observed. In the subsequent chapters, we will therefore dicuss the impact of particles on immune modulation, which is mediated by the specific nature of the particle as adjuvants in AIT.

## Mechanisms of Adjuvants—the Classical Case of Aluminum Hydroxide

During AIT, adjuvants are often used to modify the immunological and pharmacological efficacy of the vaccine. The use of adjuvants can reduce the required dose of allergen, consequently lowering treatment cost and increasing patient compliance (198). The first generation of adjuvants includes mineral salts such as aluminum hydroxide (alum, *i.e.*, particulates >1 μm) and calcium phosphate (*i.e.*, particles in nano-micro range), which are the prototypical and most commonly used adjuvants. Second generation adjuvants used in vaccines include TLR agonists, probiotics, small molecules and particulate systems, which aim to induce a shift in the already established immune response toward a Th1- and Treg-dominated activity (199). Even though adjuvants have been used in allergy vaccines since the early 20th century, the full extent of their mechanisms of action still remains to be elucidated.

### Improvement of Allergen Uptake

Adjuvants like alum, which essentially consist of particles, can *per se* improve the cellular uptake of the allergen by either acting as a delivery vehicle or by helping to efficiently target APCs. Gupta et al. (200) report on the mechanism by which alum-adsorbed antigens are readily phagocytosed by DCs, hence increasing the uptake of antigen. Furthermore, NPs have been shown to improve the uptake of antigen in bone-marrow derived cells (BMDCs) (201), which is attributed to the small size and large surface area of NPs contributing to their easy capture and internalization (202).

### Augmenting Immunogenicity

One of the main functions of adjuvants is enhancing the immunogenicity of the administered allergen, *i.e.*, to deviate the immune response from Th2- to a Th1- and Treg-dominated milieu, which can be measured by the resistance to endolysosomal proteolysis (203), upregulation of cytokines such as IL-10 (204), IL-12, IFN-γ, and downregulation of cytokines IL-4, IL-5, IL-13 (205). Furthermore, small molecules like vitamin D3 (206) and aspirin (207) used as adjuvants, have been reported to induce Treg cells. Similarly, alum initiates a Th1 response by the activation of the NLRP3 inflammasome, which further induces the production of IgG antibodies (208). TLR4 agonists such as monophosphoryl lipid A have been found to exhibit a strong potential to induce allergen-specific IgG antibodies (209). Moreover, biodegradable PLG NPs have been shown to enhance antigen-specific immune tolerance without the induction of a Th2 response (210).

### Reduction of Antigen Dose/Number of Immunizations Needed for Protective Immunity

AIT doses can be decreased if the adjuvant-associated allergen forms a depot at the site of administration, leading to its release in a controlled fashion. Alum, as well as various particulate delivery systems have been shown to form such depots and deliver antigens over a long period of time. This depot effect prolongs and sustains the allergen-specific antibody titres and, thus, can enhance the antigen uptake and presentation (211, 212).

Although many adjuvants have been developed for vaccines, only a few have been extensively considered for AIT (213). Marketed allergy vaccines usually contain adjuvants like alum, calcium phosphate, microcrystalline tyrosine, and monophosphoryl lipid A. Table 1 lists various adjuvants used in AIT.

**Table 1:** Overview on formulations in currently marketed SCIT vaccines in Europe.

**Product**	**Allergens**	**Adjuvant**	**Manufacturer**	**Reference**
**NON-MODIFIED ALLERGEN PREPARATION**				
Alutard SQ®	Pollen, HDM, animal epithelia, insect venom	Alum	ALK-Abello	(214)
Depot-HAL® F.I.T.	Pollen, HDM, animal epithelia, molds	Alum	Hal Allergy	https://www.hal-allergy.com
Novo-Helisen®Depot	Pollen, HDM, animal epithelia, molds	Alum	Allergopharma	https://compendium.ch/mpro/mnr/1072/html https://www.allergopharma.com/
Pangramin®Depot A Plus B	Pollen, HDM	Alum	Alk-Abello	https://www.alk.de
Tyro-SIT	Pollen, HDM, animal epithelia, molds	Microcrystalline tyrosine	Bencard Allergie GmbH	https://www.bencard.com
Venomenhal®	Insect venom	–	Hal Allergy	https://www.hal-allergy.com
**CHEMICALLY MODIFIED ALLERGEN PREPARATION**				
Acaroid®	HDM	Alum	Allergopharma	https://clinicaltrials.gov/ct2/show/NCT00263640
Acarovac®	HDM	Monophosphoryl lipid A	Bencard Allergie GmbH	https://www.bencard.com
Allergovit ®	Pollen	Alum	Allergopharma	https://clinicaltrials.gov/ct2/show/NCT00263601
Alustal®	Pollen, HDM, animal epithelia, molds	Alum	Stallergenes	(215)
Alutek®	Pollen, HDM, animal epithelia	Alum	Inmunotek	https://www.inmunotek.com
Alxoid®	Pollen, HDM, animal epithelia	Alum	Inmunotek	https://www.inmunotek.com
Clustoid®	Pollen, HDM, animal epithelia	Alum	Inmunotek	https://www.inmunotek.com
Clustoid®	Pollen, HDM	Alum	Roxall	(216)
Depigoid®	Pollen, HDM	Alum	Leti Pharma GmbH	https://alergia.leti.com
Phostal®	Pollen, HDM, animal epithelia, molds	Alum	Stallergenes	(215)
Pollinex Quattro®	Pollen	Monophosphoryl lipid A	Bencard Allergie GmbH	(217)

Alum is approved for a wide spectrum of human vaccines and has a long history of use, particularly in subcutaneous immunotherapy (SCIT) (199). It is simple in preparation, has a good stability and has been shown to enhance the immunogenicity of allergens. During AIT, allergens are strongly adsorbed onto the surface of alum either through electrostatic interactions, ligand exchange or hydrophilic-hydrophobic interactions, which lead to the formation of particulate matter (218). This particulate matter can then be easily phagocytosed by the APCs at the injection site, commencing the immune reaction. Most alum preparations contain small crystalline structures, which can destabilize lysozymes upon phagocytosis by inducing the secretion of agents such as heat shock protein (HSP)-70 (219), cathepsin (220), and potassium ions (221) into the cytosol. This prompts the activation of the NLRP3 inflammasome, resulting in the secretion of pro-inflammatory cytokines and the subsequent production of allergen-specific antibodies (220). Alum has furthermore been shown to induce the release of self-DNA, leading to cytotoxicity at the site of administration (222). This release of self DNA can activate either and IRF3-dependent or IRF3-independent pathway, resulting in the production of allergen-specific IgG or IgE antibodies (217).

### Unwanted Side Effects Associated With Alum in AIT

While alum is generally well-tolerated in small amounts, AIT treatments are lengthy and require frequent vaccinations (up to 16 injections within the first year of treatment) (223). Thus, there is a greater chance of developing certain adverse reactions, such as urticaria, myalgia, chronic fatigue and cognitive dysfunction in susceptible individuals. There is also an increased probability for the accumulation of aluminum salts at the site of administration, which can lead to macrophagic myofascitis (224). In some cases, alum has furthermore been reported to induce a Th2-biased immune response (225, 226) and can, thus, counteract the therapeutic mechanism of AIT.

Only a small number of studies have so far reported on the toxicity and adverse reaction of alum in immunotherapy. Hence, there is still a huge gap of knowledge, regarding the safety, toxicity, and mode of action of aluminum-based adjuvants in immunotherapy.

## Nanoparticles—an Alternative Adjuvant to Alum

**Figure 4:**
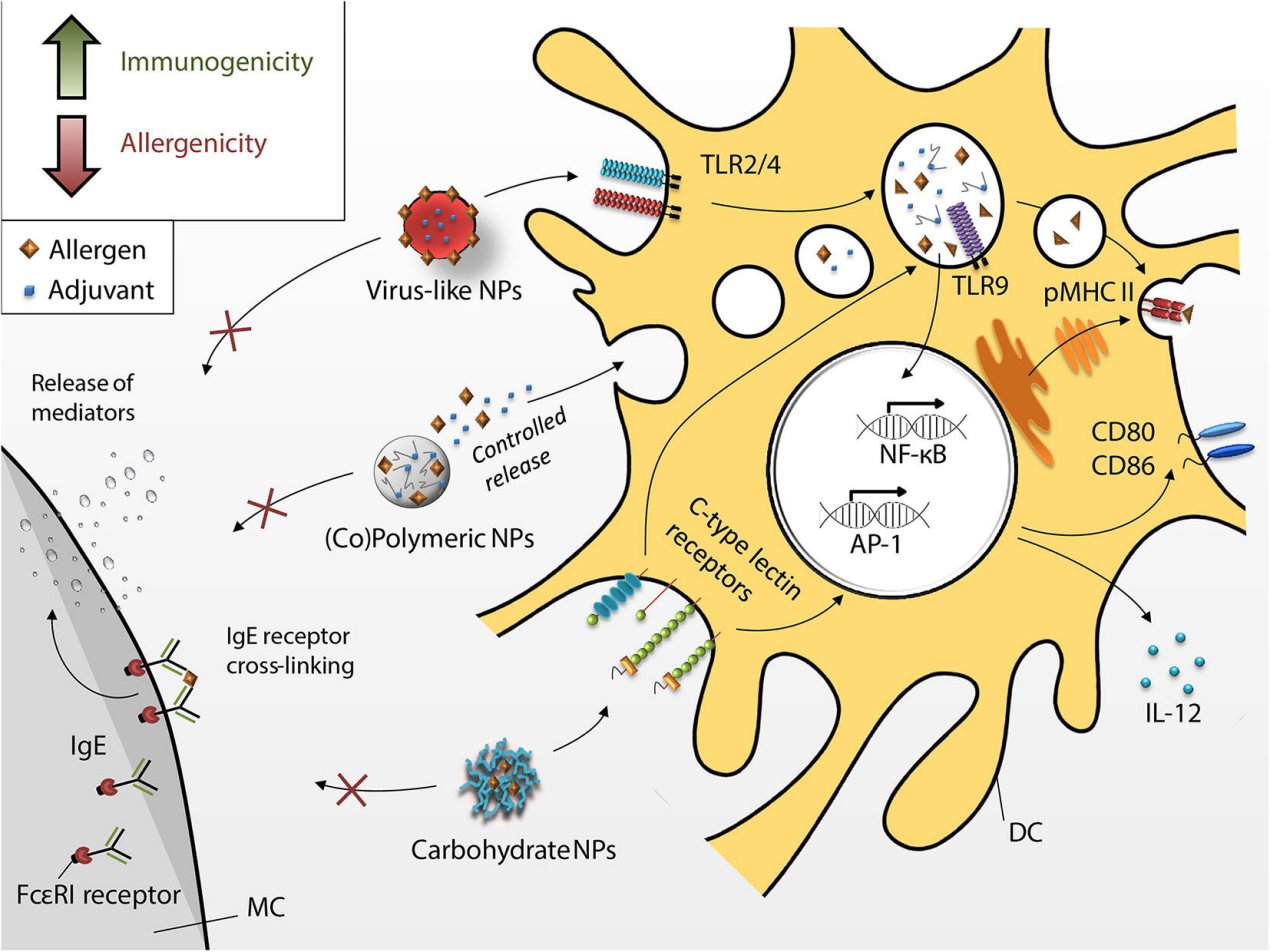
Dendritic cell-targeting by different nanoparticles. Novel approaches intend to increase immunogenicity (green arrow in box) and decrease allergenicity (red arrow in box) of the nano-fromulated vaccines (i.e., virus-like NPs, copolymer, and peptide-based polymeric NPs and carbohydrate NPs) taken up by DCs through receptor-mediated phagocytosis. Allergen encapsulation and increased uptake shall render vaccines hypoallergenic (i.e., inhibition of MC degranulation, red arrow in box) and more immunogenic (green arrow in box), hence, improving the overall efficacy of AIT.

Targeting APC with allergens incorporated into or introduced on the surface of NPs is an alternative approach to the use of alum-based adjuvants for AIT (Figure 4). Due to their size, NPs are efficiently taken up at the site of immunization. Additionally, APCs have a variety of receptors on the cell surface and their targeting orchestrates the cell activation status and the later immune polarization. Therefore, implementation of specific receptor ligands (*e.g.*, carbohydrates) into vaccine delivery systems, may not only facilitate internalization, but also modulate the subsequent immune response.

Resident skin dendritic cells form different subsets based on expression of surface receptors. Moreover, targeting of specific receptors leads to specific DCs activation determining T cell function. For example, activated epidermal Langerin^+^ DCs promote cytotoxic immune response and can be targeted *via* DEC-205. Human dermal CD1a^+^ DCs express MGL (macrophage galactose-type C-type lectin), MR (mannose receptor), DEC-205 and DC-SIGN. Targeting of these receptors activates DCs driving CD4^+^ T cell proliferation (227, 228). CD14^+^ dermal DCs express high levels of DC-SIGN and are important for generation of follicular Th cells and hence efficient antibody production. Oral administration of vaccines can target gut mucosal DCs. Depending on the environment, these CD103^+^ DCs can be either tolergenic or pro-inflammatory under inflammatory gut conditions (229). CD103^+^ CD11b^+^ have also been shown to be critical for Th17 induction (230). Thus, tailor made immune responses can be induced by targeting specific DC subsets *via* their respective receptors. A more detailed discussion on the link between DCs and T cell functions can be found elsewhere (231).

Nanomaterials are convenient systems for the introduction of functional modifications and are able to combine antigenic and adjuvant properties.

Due to their different physical and chemical properties, NPs have the ability to improve the efficacy of AIT. Here we discuss different particle types currently in either the preclinical or clinical testing phase (*e.g.*, glycoconjugates and virus-like particles) and their potential for immunotherapy.

### Carbohydrate Nanoparticles

Polysaccharides are components of fungal and bacterial cell walls and are recognized by receptors on APCs, which in turn induce strong immune responses against these pathogens. Allergy vaccines containing carbohydrates—ligands of innate-immune receptors—are more efficiently taken up by APCs, not only due to their receptor-mediated internalization, but also their particulate nature. Additionally, *via* specific receptor targeting, allergen carbohydrate NPs induce stronger responses compared to native proteins and can modulate the immune polarization.

Chitosan NPs have been shown to induce Th1 responses when administered *via* different routes. In prophylactic mouse experiments, oral immunizations with chitosan and plasmid DNA encoding the allergen induced a Th1 response and protected from subsequent allergen challenge. Chitosan particles mainly accumulated in Peyer's patches and likely were taken up by M cells (232, 233). Intranasal and oral therapeutic administrations of allergen-chitosan particles furthermore improved lung function in sensitized mice (201, 234). Similarly, sublingual allergen-maltodextrin formulations reduced AHR and Th2 responses in OVA-allergic mice (235). The authors suggest that oral Langerhans-like dendritic cells internalize the modified allergen and subsequently prime T lymphocytes in cervical lymph nodes.

Allergen neoglycoconjugates containing mannan were found to render the vaccine hypoallergenic, and to induce mixed Th1/17 immune responses after epicutaneous immunization. Bet v 1 neoglycoconjugates were tested in human skin explants and preferentially activated CD14^+^, CD14^+^ CD1a^+^ and CD14^−^ CD1a^−^ DC subsets as well as Langerhans cells. As CD14^+^ DCs overexpress mannose receptor, conjugates were most likely internalized via this C-type lectin receptor. Additionally, Bet v 1-mannan activated complement, which can enhance uptake via complement receptors (236). Animals treated intradermally with the mannan-modified allergen papain displayed significantly higher antigen-specific IgG titers in sera and showed the lowest induction of IgE responses compared to unconjugated allergen. MHC II^high^ CD8α^+^ DCs were efficiently targeted by these conjugates (237). Mannose glycodendropeptide nanostructures conjugated with Pru p 3 peptide protected sensitized mice from anaphylactic shock after sublingual applications (238). The protection was provided due to a decreased Th2 and increased Th1/Treg responses. Subcutaneous injections of allergoid-mannan conjugates for the treatment of canine atopic dermatitis resulted in a clear clinical improvement of the disease. The conjugates were shown to be internalized by human monocyte-derived dendritic cells *via* C-type lectin receptors (239, 240). Mannan neoglycoconjugates and allergoids of grass pollen and mites are currently in phase II clinical trials of sublingual and subcutaneous immunotherapy (241).

### Copolymer Nanoparticles

Encapsulating allergen in biodegradable polymeric NPs provides a better safety profile during immunizations and activates the uptake by APCs, thus enhancing cellular and humoral responses (242). Poly(lactic-co-glycolic acid) (PLGA)-based drugs are approved by the FDA and the European Medical Agency for subcutaneous, intramuscular and oral administration. In a model of murine allergic rhinitis induced by the pollen allergen Che a 3, animals were treated with recombinant Che a 3 incorporated into PLGA NPs (243, 244). In both studies, sublingual AIT with PLGA NPs resulted in the eradication of allergic rhinitis symptoms and induced Th1/Treg responses. Liu et al. (245) targeted liver sinusoidal endothelial cells with surface-modified PLGA NPs to induce tolerance against ovalbumin in a murine asthma model. In a murine cow's milk allergy model, prophylactic oral applications of beta-lactoglobulin-derived peptides incorporated into PLGA NPs induced tolerance to the whole whey protein after sensitization (242). In SLIT nanoparticles are captured within sublingual mucosa by Langerhans-like dendritic cells (246) and swallowed allergen is later taken up by M cells in Peyer's patches (244).

Polyanhydride is a copolymer of methyl vinyl and maleic anhydride (PVMA) and it is marketed as Gantrez®. The material is biocompatible and provides sustained release (247). When used in oral administrations, it can protect the allergen from enzymatic degradation and prolong the duration of allergen contact with the mucosa (199). Furthermore, PVMA carriers have been shown to target TLR2- and TLR4-inducing DC maturation and Th1 induction (248). In two recent studies, sensitized mice were treated with peanut extract encapsulated in Gantrez® NPs (199, 249). Treated mice displayed higher survival rates after allergen challenge compared to non-treated and allergen extract-treated groups. Another nut-related allergy study used cashew allergen-loaded polyanhydride NPs for oral immunizations. After a single dose, NPs induced a higher Th1/Th2 ratio and increased Treg cell count. Likely, polyanhydride nanoparticles interacted with immune cells within Peyer's patches (249).

### Peptide-Based Polymeric Nanoparticles

γ-PGA is a bacterial exopolymer, which is used with L-phenylalanine ethylester to generate self-assembling NPs. The carriers are biodegradable and due to their nature, directly target DCs and induce APC maturation. After *i.v*. injection, nanoparticle internalization was highest in CD11c^+^ and CD11c^+^ CD8^+^ splenic DCs (250). γ-PGA NPs loaded with recombinant Phl p 5 expanded allergen-specific IL-10-producing memory T cells when incubated with human monocyte-derived DCs. Nanoparticles were preferentially internalized by myeloid DCs (mDCs), but not plasmacytoid DCs. TLR2 and TLR4 played an important role in the maturation of mDCs induced by γ-PGA NPs (251). The authors of this study suggested the use of γ-PGA NPs also for intranasal immunotherapy. However, oral administration should be considered with caution, since γ-PGA-containing foods (*e.g.*, fermented soybeans) may cause late-onset anaphylaxis (252).

Protamine is a 4 kDa-cationic peptide, which is commonly used to design carriers for cancer therapy (253, 254). Pali-Scholl et al. (255) used protamine NPs doped with the TLR9 ligands CpG-oligodeoxynucleotides, so-called proticles, for anti-allergy immunizations. Proticles were efficiently taken up by BMDCs and activated them. *In vivo*, proticles formed a depot at the injection site and subcutaneous immunotherapy of peanut-allergic mice resulted in a modulation of the immune responses toward Th1.

### Virus-Like Nanoparticles

Virus-like nanoparticles (VLP) are derived from viral capsid proteins and are often used for allergen-specific immunotherapy in conjunction with adjuvants such as CpG oligodeoxynucleotides (256), or more recently, tetanus epitopes (257). A series of clinical studies with allergen extracts and CpG oligodeoxynucleotides in bacteriophage QβG10 coat proteins (CYT003) were performed by Cytos Biotechnology (258, 259). The vaccine was well-tolerated by sensitized individuals without any severe adverse effects and alleviated allergic symptoms after 10 weeks of AIT. However, in later studies the company focused on unspecific treatment of asthma with CYT003 (without allergen extract) and failed to demonstrate efficacy in a phase II clinical trial (260). Kratzer et al. (261) delivered Art v 1 allergen packed in a VLP-envelope from Moloney murine leukemia virus (MA::Art v 1 VNP). Preventive capacity of the nanoparticles was tested in humanized mouse model of mugwort pollen allergy. After intranasal application, these VLPs targeted CD103^+^ DCs in lung and alveolar macrophages resulting in Th1/Treg responses that had a protective effect on subsequent sensitization with mugwort pollen extract.

### Inorganic Nanoparticles

Inorganic NPs based on silicon dioxide carriers have displayed potential for AIT. They are physically and thermally stable, can have a wide range of possible chemical modifications on their surface, and can be produced in a size range of 3 to several hundred nanometers (262). Silica NPs can either have a solid core with a functionalized surface, or be mesoporous, *i.e.*, have the ability to adsorb protein internally and may provide sustained release of the antigen (263). Mesoporous silica NPs associated with Der f 2 have been studied in a murine HDM allergy model. Subcutaneous injections have shown a preventive effect with a decreased Th2 response and boosted Th1 immunity with elevated allergen-specific IgG levels (264). However, safety of inorganic nanoparticles is still under debate and this may prevent implementation of this method in the clinic (265).

### Clinical Outlook

Chitosan, γ-PGA nanoparticles and proticles were successfully tested in animal models, but no new studies have been published in the last 7 years. PLGA- and PVMA-based allergen-specific immunotherapy has shown positive results in pre-clinical animal models in the last 2 years. However, no data on ongoing clinical studies is available. Two types of nanoparticles which made their way to the clinics are mannan conjugates and VLPs. Clinical trials with empty VLPs did not show any efficacy and were discontinued. New types of VLPs, MA::Art v 1, have not been tested in the clinics yet.

Subcutaneous and sublingual administration of hypoallergenic mannan glycoconjugates were efficient in animal models of allergic sensitization and currently are in phase 2 clinical trials (241). Preclinical studies with carbohydrate nanoparticles have shown that this approach can be further improved by the use of skin as immunization site. In contrast to hypodermis, where subcutaneous vaccinations are performed, epidermis and dermis are rich in APCs. *I.d*. injection or epicutaneous application via laser-generated micropores (189) allows direct activation of dermal DCs by nanoparticles (237). The synergy of particulate allergen conjugates targeting C-type lectin receptors on dermal DCs with delivery to superficial skin layers (236) may greatly improve existing approaches of AIT. Additionally, substitution of *s.c*. injections with epicutaneous application and shorter treatment protocols may improve patient compliance for anti-allergic immunizations.

## Concluding Remarks

Much has been learned about the properties of individual allergens and about general factors that are associated with increased prevalence of allergic diseases. Still, it is not understood why a particular person develops sensitivity against a specific allergen. Allergy is a multifactorial disease and its induction involves bystander factors, among which particles play an important role: They promote uptake into cells, can carry a multitude of chemicals as cargo and offer a platform to achieve high local concentrations of effectors. Understanding the complex interplay between particles and allergens will be essential for fully elucidating the genesis of allergy as well as for developing new generations of therapeutics.

## Author Contributions

All authors were involved in concept drafting, literature screening, design of display items, writing, and editing of the article.

## Conflict of Interest

The authors declare that the research was conducted in the absence of any commercial or financial relationships that could be construed as a potential conflict of interest.
