# Erratum: Mechanisms of Particles in Sensitization, Effector Function and Therapy of Allergic Disease

**DOI:** 10.3389/fimmu.2022.891445

**Published:** 2022-03-23

**Authors:** Frontiers Production Office

**Affiliations:** Frontiers Media SA, Lausanne, Switzerland

**Keywords:** adjuvants, alum, animal dander, house dust mite feces, immunomodulation, mold spores, nanomedicine, pollen

Due to a production error, the first author’s name was incorrectly written as “Anna I. Joubert”. The correct name is “Isabella Anna Joubert”. The publisher apologizes for this mistake.

The original version of this article has been updated.

